# Biochemical classification of tauopathies by immunoblot, protein sequence and mass spectrometric analyses of sarkosyl-insoluble and trypsin-resistant tau

**DOI:** 10.1007/s00401-015-1503-3

**Published:** 2015-11-04

**Authors:** Sayuri Taniguchi-Watanabe, Tetsuaki Arai, Fuyuki Kametani, Takashi Nonaka, Masami Masuda-Suzukake, Airi Tarutani, Shigeo Murayama, Yuko Saito, Kunimasa Arima, Mari Yoshida, Haruhiko Akiyama, Andrew Robinson, David M. A. Mann, Takeshi Iwatsubo, Masato Hasegawa

**Affiliations:** Department of Dementia and Higher Brain Function, Tokyo Metropolitan Institute of Medical Science, 2-1-6 Kamikitazawa, Setagaya-ku, Tokyo, 156-8506 Japan; Department of Neuropathology, The University of Tokyo, 7-3-1 Hongo, Bunkyo-ku, Tokyo, 113-8654 Japan; Department of Psychiatry, Graduate School of Comprehensive Human Sciences, University of Tsukuba, 2-1-1 Amakubo, Ibaraki, Tsukuba, 305-8576 Japan; Department of Neuropathology, Tokyo Metropolitan Institute of Gerontology, 35-2 Sakaecho, Itabashi-ku, Tokyo, 173-0015 Japan; Department of Pathology and Laboratory Medicine, National Center Hospital, National Center of Neurology and Psychiatry, 4-1-1 Ogawahigashi, Kodaira, Tokyo, 187-8551 Japan; Institute for Medical Science of Aging, Aichi Medical University, 1-1 Yazakokarimata, Nagakute, Aichi, 480-1195 Japan; Institute of Brain, Behaviour and Mental Health, Clinical and Cognitive Neuroscience Research Group, University of Manchester, Salford Royal Hospital, Salford, M6 8HD UK

**Keywords:** Tau, Alzheimer, Pick, CBD, PSP, *MAPT*, Strains

## Abstract

Intracellular filamentous tau pathology is the defining feature of tauopathies, which form a subset of neurodegenerative diseases. We have analyzed pathological tau in Alzheimer’s disease, and in frontotemporal lobar degeneration associated with tauopathy to include cases with Pick bodies, corticobasal degeneration, progressive supranuclear palsy, and ones due to intronic mutations in *MAPT*. We found that the C-terminal band pattern of the pathological tau species is distinct for each disease. Immunoblot analysis of trypsin-resistant tau indicated that the different band patterns of the 7–18 kDa fragments in these diseases likely reflect different conformations of tau molecular species. Protein sequence and mass spectrometric analyses revealed the carboxyl-terminal region (residues 243–406) of tau comprises the protease-resistant core units of the tau aggregates, and the sequence lengths and precise regions involved are different among the diseases. These unique assembled tau cores may be used to classify and diagnose disease strains. Based on these results, we propose a new clinicopathological classification of tauopathies based on the biochemical properties of tau.

**Electronic supplementary material:**

The online version of this article (doi:10.1007/s00401-015-1503-3) contains supplementary material, which is available to authorized users.

## Introduction

Tauopathies are sporadic or familial neurodegenerative diseases characterized by intracytoplasmic aggregates of hyperphosphorylated abnormal tau protein [6, 15, 23]. Most cases of Alzheimer’s disease (AD), and ones of frontotemporal lobar degeneration associated with tauopathy (FTLD-tau), including cases with Pick bodies (PiD), corticobasal degeneration (CBD), progressive supranuclear palsy (PSP) and argyrophilic grain disease (AGD), are sporadic. The morphology and regional distribution of pathology differ among these diseases, with characteristic tau pathologies being observed in degenerating neuronal and/or glial cells in each disease. These include neurofibrillary tangles (NFTs) in AD, Pick bodies in PiD, astrocytic plaques in CBD, tuft-shaped astrocytes in PSP, and argyrophilic grains in AGD. Various tau pathologies have also been reported in familial FTLD-tau cases caused by exonic or intronic mutations in the *MAPT* gene. They are neurofibrillary-like pathologies in cases with the M337V and R406W mutations, Pick body-like tau structures in cases with the G272V, S320F, K369I, and G389R mutations, and PSP/CBD-like glial-dominant tau pathologies in most cases with missense or intron mutations in exon10/intron10 [14, 34]. Ultrastructurally, these pathological inclusions consist of fibrils or filaments 10–25 nm diameter, composed of hyperphosphorylated tau. Reported structures include paired helical filaments (PHFs) (10–20 nm) in AD, straight filaments (15–18 nm) in PiD, straight filaments (13–14 nm) in PSP, twisted filaments (20 nm) in CBD, and twisted ribbons (15–25 nm) in cases with *MAPT* intronic mutations [4, 14]. These abnormal forms of tau are highly insoluble in those non-ionic and ionic detergents usually used for biochemical studies, such as Triton-X100 and sarkosyl, and can be enriched by making use of this insolubility. Biochemical analyses of sarkosyl-insoluble tau have demonstrated that the abnormal tau is hyperphosphorylated at more than 20 sites near the microtubule-binding repeat region and partially ubiquitinated in the repeat region [17, 27]. In AD, both 3-repeat (3R) and 4-repeat (4R) tau isoforms are accumulated as filamentous aggregates (PHFs) [16], whereas only 3R tau isoforms are deposited in PiD [10], and 4R tau isoforms are primarily accumulated in PSP and CBD [2, 32, 37]. It has also been reported that only 4R tau isoforms are deposited in *MAPT* intronic mutations that increase the relative expression ratios of 4R tau isoforms. Importantly, both CBD and PSP are 4R-tauopathies, but they can be biochemically distinguished by the banding pattern of the C-terminal fragments of tau [1]. Thus, the involvement of the C-terminal region containing the microtubule-binding domains is critical in determining the neuropathology of tauopathies. As for other post-translational modifications, deamidation and racemization of asparagine residues have been reported in AD-tau, and the deamidation of Asn279 (located in the epitope of monoclonal antibody RD4) differs between AD and 4R tauopathies [9, 40]. Ser 262 is less phosphorylated in PiD, but is similarly phosphorylated in the other tauopathies [3, 31]. Using immunoelectron microscopy of PHFs, with or without protease treatments, the antiparallel assembly model and the structural stability of the microtubule-binding domains have been suggested [5, 22, 41]. In vitro studies also showed that recombinant tau fragments containing the microtubule-binding domains, assembled into filaments by a β-sheet-like interaction [38]. Furthermore, recent studies have shown that tau fibrils made from full-length tau or the microtubule-binding domains can internalize and induce tau fibril formation in cultured cells [11, 29]. Although the abnormal tau species in tauopathies share many immunochemical and biochemical features, little is known of the structural differences in abnormal tau species among the diseases. It is also unknown why these tau pathologies are so diverse in each disease, but relatively homogeneous in individual patients, or how they develop. A growing body of evidence suggests that intracellular abnormal proteins, including tau, have prion-like properties, i.e., they can convert normal proteins into an abnormal form which can be transmitted from cell to cell and thereby propagated throughout the brain [8, 13, 28, 29]. The pattern of spread of these neuronal and glial intracellular abnormal protein lesions and the progressive nature of the conditions can be well explained by such prion-like propagation of these proteins. Here we show that, as in prion disease, the protease-resistant tau banding patterns, covering the carboxy-terminal region (243–406), are different between the diseases, and may be useful in the biochemical classification of tauopathies.

## Materials and methods

### Brain tissues

The subjects in this study included five patients with PiD, nine patients with PSP, eight patients with CBD, one atypical case with pathological features of both CBD and PSP, eight patients with intronic *MAPT* mutations (7 patients with +16 and 1 patient with +13 mutation) and ten patients with AD. The age, sex, disease duration, brain weight, postmortem interval, and brain regions examined are given in Table 1 (cases no 1~27 used in the first study and additional cases no 28~40 are listed). Frozen brain tissues of tauopathies were diagnosed by Neuropathologists at Manchester University, Aichi Medical University, Tokyo Metropolitan Institute of Gerontology, and National Center Hospital, National Center of Neurology and Psychiatry. The frozen tissues of all subjects were stored at −80 °C until analysis.

**Table 1:** Description of the cases used in the initial study (no 1~27), and the additional cases used in the second study (no 28~40)

Diagnosis	Case no.	Sex	Age at death (year)	Duration (year)	Brain (g)	PMI (h)
PiD	1	F	62	10	928	N/A
PiD	2	M	56	10	1150	N/A
PSP	3	F	N/A	N/A	N/A	N/A
PSP	4	F	71	7	1070	8
PSP	18	M	74	10	1265	N/A
PSP	24	M	72	7	1290	N/A
PSP	25	F	68	9	1250	N/A
PSP	26	M	80	7	1060	N/A
CBD	5	F	74	N/A	1010	7
CBD	6	F	83	27	1024	8
CBD	7	F	80	10	990	10
CBD	17	F	65	5	950	2
CBD	23	M	71	6	1250	0.5
CBD + PSP	19	M	67	4	840	7
*MAPT* (+16)	8	M	55	12	1240	N/A
*MAPT* (+16)	9	F	65	13	1040	N/A
*MAPT* (+16)	10	M	46	53	1240	18
*MAPT* (+13)	11	M	70	5	1100	18
*MAPT* (+16)	27	M	65	1	N/A	24
AD	12	M	72	5	1240	2
AD	13	F	72	6	1100	4
AD	14	F	68	20	775	13
AD	15	M	80	N/A	1390	7
AD	16	F	70	N/A	1126	7
AD	20	F	65	9	1165	N/A
AD	21	F	76	13	950	N/A
AD	22	M	56	4	1350	7
PiD	28	F	58	8	1065	N/A
PiD	29	M	77	4	N/A	N/A
PiD	30	M	69	10	850	N/A
PSP	31	M	88	6	1038	4.4
PSP	32	M	85	15	850	1
PSP	33	M	87	8	1120	15
CBD	34	F	87	4	1026	36
CBD	35	M	88	7	1025	15
CBD	36	F	71	8	1020	7.5
*MAPT* (+16)	37	F	63	15	1100	96
*MAPT* (+16)	38	M	66	N/A	N/A	N/A
AD	39	M	72	12	1251	N/A
AD	40	M	62	8	1100	24

*PMI* postmortem interval, *PiD* Pick’s disease, *PSP* progressive supranuclear palsy, *CBD* corticobasal degeneration, *MAPT (+16)* cases with *MAPT* intron +16 mutation, *AD* Alzheimer’s disease, *N/A* not available

### Antibodies used in this study

Antibodies used in this study were anti-tau T46 (Thermo Scientific), AT180 (Thermo Scientific), 12E8 [33] (a gift from Elan Pharmaceuticals, TauC4 [21] (a gift from Dr. Ihara), PHF1 (a gift from Dr. Davies), pS409 and AP422 [27] (gifts from Dr. Ihara). The epitopes of these antibodies are illustrated in Fig. 2d. The new TauC4 antibody was raised against synthetic peptide of IGSLDNITHVPGGGNK (corresponding to residues 354–369 of the 441 human tau isoform) with cysteine at the N-terminus conjugated with KLH. The new TauC4 and 12E8 antibodies were characterized by immunoblot analysis as shown in suppl Fig. 1a, b.

**Fig. 1:**
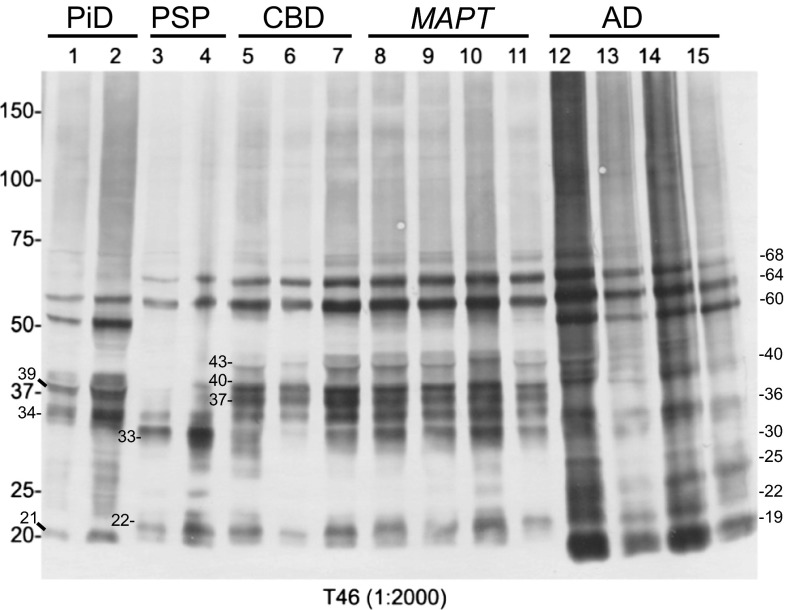
Immunoblot analysis of sarkosyl-insoluble tau in tauopathies. Hyperphosphorylated full-length tau (60, 64 and 68 kDa), various C-terminal fragments and smears were detected in the sarkosyl-insoluble fractions of tauopathy brains by T46 (anti-tau C-terminus). The banding patterns are characteristic for each disease, although the patterns of fragments of CBD and *MAPT* (intron mutation) seem very similar

### Preparation of sarkosyl-insoluble tau

For detection of abnormal tau accumulated in brains of patients, the sarkosyl-insoluble pellet was prepared as follows. Frozen brain tissue (0.2–0.5 g) was homogenized in 20 volumes (v/w) of extraction buffer containing 10 mM Tris–HCl (pH 7.5), 0.8 M NaCl, 10 % sucrose, 1 mM EGTA, 2 % sarkosyl and incubated for 30 min at 37 °C. After centrifugation at 20,000×*g* for 10 min at 25 °C, the supernatants were taken, transferred to 1.5 mL tubes and ultracentrifuged at 100,000×*g* for 20 min at 25 °C. The pellets were washed by ultracentrifugation with 0.5 mL of sterile saline, solubilized in SDS-sample buffer and subjected to 4–20 % gradient polyacrylamide gel (Wako) SDS-PAGE. Proteins were transferred to PVDF membrane, incubated overnight with the anti-tau monoclonal antibody T46, biotinylated 2nd antibody, avidin–biotin complex (Vector) and developed with diaminobenzidine and nickel chloride. For preparation of trypsin-resistant tau, brain tissue (0.2–0.5 g) was homogenized and extracted sequentially with 5 volumes (v/w) of extraction buffer, buffer containing 1 % Triton-X100, and buffer containing 1 % sarkosyl. After incubation in 1 % sarkosyl, the sample was centrifuged at 20,000×*g* for 20 min at 25 °C. The supernatant was ultracentrifuged at 453,000×*g* for 20 min at 25 °C, and the sarkosyl-insoluble pellet was subjected to trypsin treatment.

### In-gel digestion and nano-LC/MS/MS analysis

Sarkosyl-insoluble fractions were run on 4–20 % gradient gels and separated proteins corresponding to the fragments were excised and soaked in 50 mM Tris–HCl, pH 8.0 containing 50 % acetonitrile for 30 min. The gels were dried in a Speed-Vac (Savant) and incubated in 50 mM Tris–HCl, pH 8.0 containing 125–250 ng of modified trypsin, Lys-C (Roche Diagnostics) or V8 (Sigma) at 37 °C for 14–20 h. The digests were extracted from the gel, evaporated in a Speed-Vac, resuspended in 0.1 % formic acid containing 2 % acetonitrile and subjected to a DiNa HPLC system fitted with an automatic sampler (KYA technology). A packed nano-capillary column NTCC-360/75-3-123 (0.075 mm I.D. × 125 mm L, particle diameter 3 µm, Nikkyo Technos) was used at a flow rate of 300 nl/min with a 2–80 % linear gradient of acetonitrile for 60 min. Eluted peptides were directly detected with an ion trap mass spectrometer, Velos Pro (Thermo Scientific). The data were analyzed with Mascot (Matrix Science) software.

### Expression and purification of recombinant wild-type and deletion mutant of human tau

Human tau cDNA (3R1N and 4R1N) in bacterial expression plasmid pRK172 were kind gifts from Dr. Michel Goedert. The deletion mutants (1-163, 1-226, 3Rtau 251-441 and 4Rtau 251-441) were constructed using PCR using 3R1N and 4R1N in pRK172 as a template. All constructs were verified by DNA sequencing. Wild-type tau and the deletion mutants were expressed in *E. coli* BL21 (DE3). Wild-type tau, 3Rtau 251–441 and 4Rtau 251–441 were extracted in 50 mM Pipes buffer (pH 6.8) containing 5 mM EGTA, 1 mM DTT and 0.5 mM PMSF, and boiled for 5 min. After centrifugation, the supernatants (heat-stable fraction) were subjected to SP-Sepharose and eluted with extraction buffer containing 0.5 M NaCl. The N-terminal tau fragments (1–163 and 1–226) were extracted in 50 mM Tris buffer (pH 9.0) containing 5 mM EGTA, 1 mM DTT and 0.5 mM PMSF, and boiled for 5 min. The supernatants were then subjected to Q-Sepharose and eluted with extraction buffer containing 0.5 M NaCl. The eluates were concentrated by 50 % saturated ammonium sulfate precipitation. The pellets were resuspended in and dialyzed against 30 mM Tris–HCl, pH 7.5.

### Trypsin digestion of sarkosyl-insoluble tau and purification of trypsin-resistant tau

Sarkosyl-insoluble pellets from 0.5 to 1 g of AD, PiD, CBD, PSP, CBD + PSP and *MAPT* cortices were suspended in 0.1 mL of 30 mM Tris (pH 7.5) containing 10 mM CaCl_2_ by sonication. Trypsin was added at a final concentration of 0.5–2 mg/mL and incubated at 37 °C for 1 h. The digestion was stopped by adding 2 % SDS for SDS-PAGE, or 6 M guanidine HCl for further purification, which was performed by size-exclusion chromatography on a TSK gel SW-3000 column [2.1 × 300 mm, TOSOH] equilibrated with 6 M guanidine HCl in 10 mM phosphate (pH 6.0) and on a reverse-phase HPLC with an Aquapore RP300 column (2.1 × 100 mm). For dephosphorylation, the sarkosyl-insoluble pellets or the trypsin-resistant cores of tau filaments were suspended in 50 mM Tris–HCl containing 6 M guanidine HCl, then dialyzed overnight at 4 °C against 50 mM Tris–HCl (pH 8.8), and centrifuged at 20,400*g* for 10 min. The supernatants were incubated with *E. coli* alkaline phosphatase (10 U/mL; type III, Sigma) at 67 °C for 2 h.

### Protein sequence and mass spectrometric analyses of trypsin-resistant tau fragments

Trypsin-resistant fragments on a PVDF membrane were sequenced on an Applied Biosystems 477A protein sequencer equipped with an online 120A PTH analyzer and/or a 473A protein sequencer. MALDI-TOF MS analysis of purified trypsin-resistant fragments was performed by a Voyager-DE Pro MALDI-TOF mass spectrometer (PerSeptive Biosystems). To determine the C-terminal sequence, the purified trypsin-resistant fragments were digested with V8 at molar ratio of 1:20 and analyzed by LC/MS/MS. LC/MS/MS was performed on an ion trap mass spectrometer, LXQ (Thermo Fisher Scientific), with a reverse-phase capillary column (Develosil ODS-HG5, 0.075 mm i.d., 50 mm or 0.075 mm i.d., 150 mm, Nomura Chemical) at a flow rate of 200 or 300 nl/min with a 4–80 % linear gradient of acetonitrile.

### Immunoelectron microscopy

Aliquots of the sarkosyl-insoluble fractions (1:5–10 diluted in DW) from tauopathy brains were placed on collodion-coated 300-mesh nickel grids. After drying, the grids were blocked with 30 mM Tris–HCl, pH 7.5 containing 2 mg/mL BSA, and incubated overnight with T46 antibody at a dilution of 1:500. The grids were rinsed and reacted with secondary antibody conjugated to 15-nm gold particles (1:50) to distinguish from ferritin particles, the major contaminants in the sarkosyl-insoluble fractions. Samples were stained with phosphotungstate and observed as described [35].

## Results

### Sarkosyl-insoluble C-terminal tau fragments in tauopathy brains

Pathological tau in the sarkosyl-insoluble fractions from brains of 15 patients with neuropathologically diagnosed tauopathy (two cases with PiD, two cases with PSP, three cases with CBD, four cases with intronic mutations in *MAPT* and four cases with AD) was investigated by immunoblot analysis. Anti-tau antibody T46, which recognizes C-terminal region of tau (404–441), detected hyperphosphorylated full-length tau and C-terminal tau fragments deposited in these brains (Fig. 1). In accordance with previous reports, with two major bands being present at 60 and 64 kDa in PiD, two major bands at 64 and 68 kDa in PSP, CBD and *MAPT*, and three bands at 60, 64 and 68 kDa in AD were detected, confirming that the band patterns of hyperphosphorylated full-length tau are useful for biochemical diagnosis of 3R and 4R tauopathies. In addition to these full-length tau banding patterns, various C-terminal fragments were characteristic for each disease. They included bands at 21, 34 and 39 kDa in PiD, bands at 22 and 33 kDa in PSP, bands at 37–40 and 43 kDa in CBD and *MAPT*, and bands at 19, 22, 25, 30, 36 and 40 kDa in AD, such findings confirming previous reports that the 33 and ~37 kDa bands are characteristic of PSP and CBD, respectively [1].

Then, we tried to identify the N-terminal sequences of these other fragments by in-gel digestion with trypsin, Lys-C and V8 proteases followed by nano-LC/MS/MS analysis. The N-terminal sequences of these fragments identified in this study are summarized in supplementary Table 1. Several different N-terminal fragments were identified from each molecular size, suggesting that the N-termini are heterogeneous. Some characteristic sequences, such as 226 VAVV and 228 VVRT, were detected in the 22–36 kD fragments of AD tau, which were not detected in other tauopathies, suggesting that AD-tau is distinct from those of other tauopathies in the C-terminal fragments. Our previous study, using protein sequencing together with immunoblot analysis, showed that the ~33 kD bands in PSP and the ~37 kD bands in CBD contain N-terminal sequences 169–175 and 187–192, respectively [1]. The peptide with N-terminal sequence 187 EPPK, which was previously identified in the ~37 kD fragments of CBD, was also detected in the 22 kD band of CBD, but not in PSP. The C-terminal processed fragments with the same N-terminus in the fraction may be detected. However, this is inconsistent with previous result in the aspect of molecular weight. This is probably due to the methods used in these studies (purified fragments were analyzed by protein sequencing in the previous study, but unpurified samples were analyzed by in-gel digestion and LC/MS/MS in this study). Thus, it is difficult to identify the N-terminal sequences of these fragments from unpurified heterogeneous samples, even if using highly sensitive LC/MS/MS analysis.

We presumed that these characteristic tau fragments may be produced by processing of full-length tau after aggregation and may represent different conformations of tau aggregates. To test this hypothesis, we investigated the protease-resistant tau fragments of these pathological tau species from tauopathy brains. To examine the structural differences of pathological tau among the diseases, we used trypsin treatment, because tau contains many Lys and Arg residues in the C-terminal region, and tryptic peptides are suitable for protein sequencing and mass spectrometric analysis. To determine the optimum concentration of trypsin, sarkosyl-insoluble fractions of AD brains were treated with 0.5–2.0 mg/mL trypsin, and digestion of tau was monitored by immunoblotting with T46 and TauC4 (Suppl Fig. 2). After treatment with 0.5–2 mg/mL trypsin for 1 h, T46-positive full-length and smeared tau had completely disappeared (Suppl Fig. 2a), but trypsin-resistant tau bands were detected at 7, 12 and 15 kDa with TauC4 (Suppl Fig. 2b). These results indicate that most of the C-terminus of tau (epitope of T46) in PHFs can be cleaved by trypsin, but the final microtubule-binding region (epitope of TauC4) is resistant to trypsin. Since the T46 epitope (404–441) is more C-terminal than that of TauC4 (354–369), it is possible that the T46 epitope is cleaved by trypsin whereas the TauC4 epitope is resistant to trypsin.

### Trypsin-resistant tau banding patterns in tauopathy brains

**Fig. 2:**
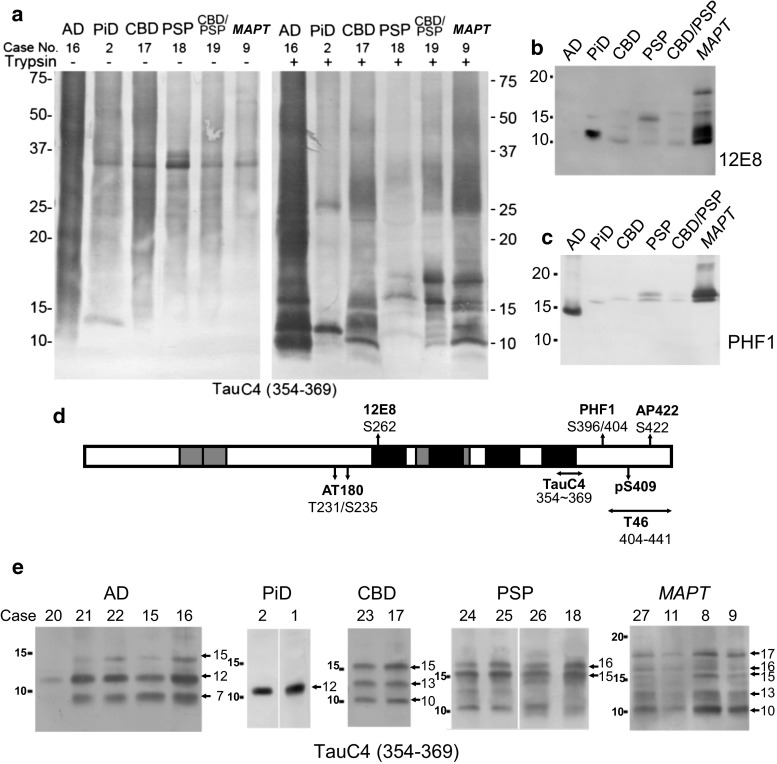
Immunoblot analysis of trypsin-resistant tau in tauopathies. **a** Immunoblot analysis of sarkosyl-insoluble tau from AD, PiD, CBD, PSP, CBD + PSP, and *MAPT* cases, before and after trypsin digestion with anti-tau 354–369 (TauC4). **b** Immunoblot analysis of trypsin-resistant tau with 12E8. **c** Immunoblot analysis of trypsin-resistant tau with PHF1. **d** Schematic illustration of epitopes of antibodies used in this study. **e** Immunoblot analysis of trypsin-resistant tau prepared from multiple cases of AD, PiD, CBD, PSP and *MAPT* cases

**Fig. 3:**
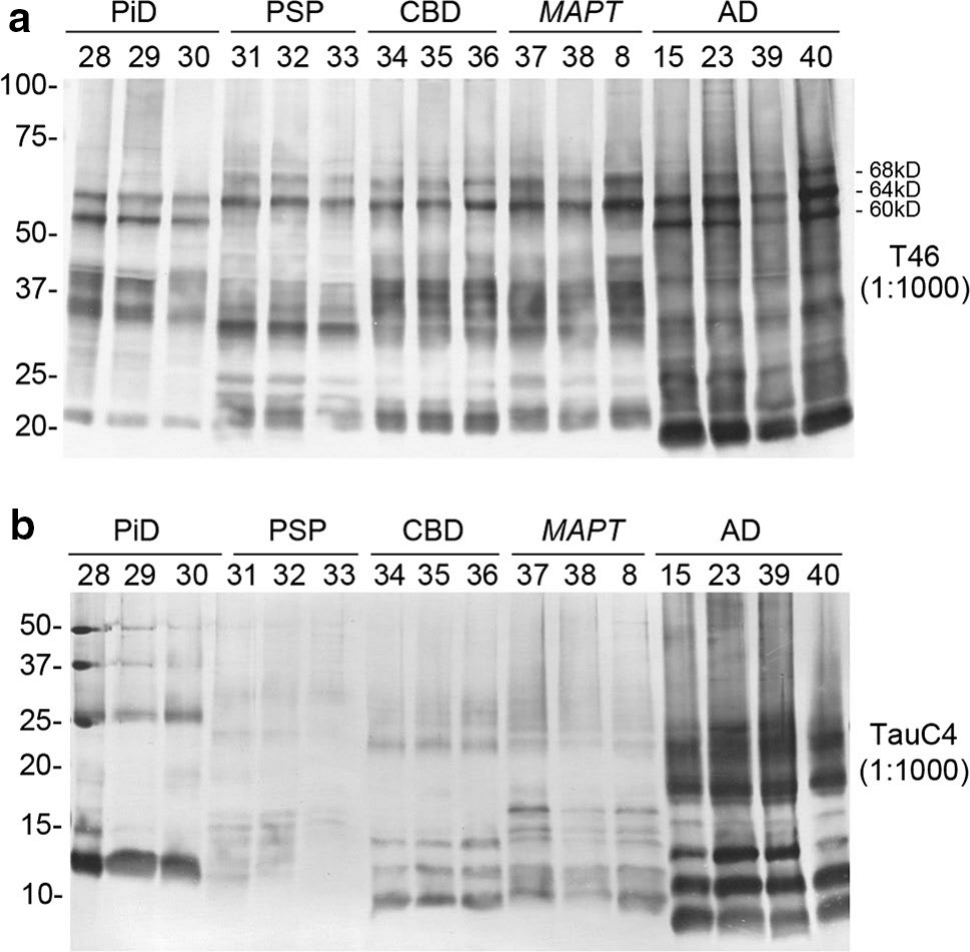
Immunoblot analyses of sarkosyl-insoluble tau and the trypsin-resistant fragments in tauopathies (additional cases). **a** Immunoblot analysis of sarkosyl-insoluble tau from three PiD, three CBD, three PSP, three *MAPT* and four AD cases. 13 additional cases (No. 28–40) were run on a gel with three cases (8, 15 and 23) analyzed in the initial study and immunoblotted with T46 antibody. **b** The trypsin-resistant tau from three PiD, three CBD, three PSP, three *MAPT* and four AD cases after the treatment of 0.1 mg/mL trypsin for 1 h were detected with TauC4 in a western blot membrane

Next, we examined the trypsin-resistant tau bands from various tauopathy brains, including AD, PiD, CBD, PSP, a combination case of CBD and PSP (CBD + PSP), and *MAPT*, by means of 1.0 mg/mL trypsin treatment followed by immunoblotting with TauC4, AT180, 12E8, PHF1, pS409 and AP422 (Fig. 2). TauC4 detected a 12 kDa band in PiD, 10, 13 and 15 kDa bands in CBD, 15, 16 kDa bands in PSP, 10, 13, 15 and 16 kDa bands in CBD + PSP, and 10, 13, 15, 16 and 17 kDa bands in *MAPT* (Fig. 2a). AT180, pS409 and AP422 did not detect any band after trypsin digestion (data not shown), but 12E8 detected a major 12 kDa band in PiD, some weak bands at 10–15 kDa in CBD, PSP and CBD + PSP, and several strong bands at 10–17 kDa in *MAPT* (Fig. 2b). The reaction of 12E8 may be due to cross-reactivity with the unphosphorylated epitope. As shown in Suppl Fig. 1b, we confirmed that 12E8 cross-reacts with unphosphorylated recombinant full-length tau and more strongly with the C-terminal fragment. In contrast, PHF1 detected a major 15 kDa band in AD, weak bands at 15–17 kDa in PiD, PSP, CBD and CBD + PSP, and doublet bands of 17 kDa in *MAPT* (Fig. 2c). The epitopes of these antibodies are illustrated in Fig. 2d. Overall, the results indicate that microtubule-binding repeat regions, including the TauC4 epitope, form the trypsin-resistant cores of pathological tau in these tauopathies, whereas the N-terminal and C-terminal regions outside the repeats are trypsin sensitive. To examine whether the trypsin-resistant tau band patterns are characteristic for each disease, we prepared sarkosyl-insoluble fractions from brains of five cases with AD, two cases with PiD, two cases with CBD, four cases with PSP and four cases with *MAPT*. Trypsin treatment followed by immunoblotting with TauC4 confirmed that each disease showed a consistent, characteristic band pattern of trypsin-resistant tau (Fig. 2e). The differences in the C-terminal banding pattern with T46 antibody and the trypsin-resistant banding pattern with TauC4 antibody were further confirmed by the analysis of additional 13 cases (three PiD, three PSP, three CBD, two *MAPT* and two AD cases) with three cases used in the first study in one western blot membrane (Fig. 3a, b).

### Protein sequence and mass spectrometric analyses of trypsin-resistant tau in tauopathy brains

**Fig. 4:**
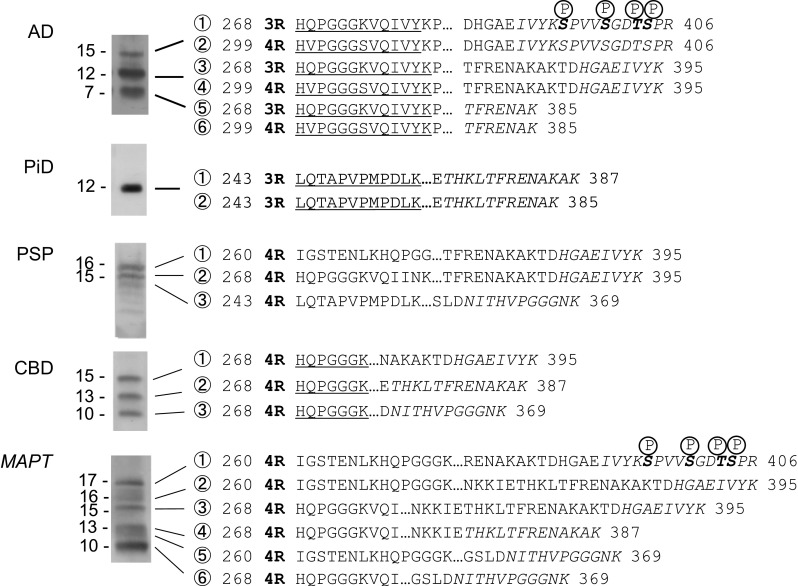
N-terminal sequences identified by protein sequencing, C-terminal peptides identified by LC/MS/MS analysis of the V8 digests, or predicted sequences by MALDI-TOF MS analysis. The results of protein sequence analysis of each band of purified trypsin-resistant tau from tauopathy brains are indicated by *underlining* and the peptides identified by LC/MS/MS analysis of V8 digestion products are indicated by *italic characters*. Possible phosphorylation sites are indicated by *bold letter* with P

To further analyze the trypsin-resistant tau, we purified trypsin-resistant tau fragments from these brains. The trypsin-resistant tau preparations were dissolved in 6 M guanidine HCl and separated by gel filtration. After dialysis and immunoblot analysis, these fragments were further purified by reverse-phase HPLC. The trypsin-resistant tau was analyzed by MALDI-TOF MS analysis and the results are summarized in Table 2. The observed mass signals were higher than expected molecular mass. These can be identified as the sodium adduct ions [M + Na]^+^. We performed mass analysis both before and after alkaline phosphatase treatment, because it has been shown that pathological tau is hyperphosphorylated, and that the phosphorylation inhibits peptide ionization. Furthermore, the N-terminal sequence of each band on the PVDF membrane was determined with a protein sequencer. The N-terminal sequences identified by protein sequencing and LC/MS/MS analysis together with MALDI-TOF MS analysis of these purified fragments are shown in Fig. 4. Phosphorylation was detected in some of these fragments, but the other post-translational modifications, such as acetylation, methylation and glycosylation were not detected in these fragments by this analysis.

**Table 2:** Summary of MALDI-TOF/MS/MS and immunoblot analyses of trypsin-resistant tau in tauopathy brains

Tauopathy	Signal no.	Measured MS (AP−)	Measured MS (AP+)	Calculated MS +Na(22)	Assignment	12E8(S262)	PHF1(pS396/404)	Apparent MW
AD	①	–	11,597.7	11,596.1	3R 268–406	–	○	15
	②	–	11,527.6	11,526.1	4R 299–406	–	○	15
	③	10,517.1	10,517.3	10,513.6	3R 268–395	–	–	12
	④	10,447.2	10,448.3	10,443.5	4R 299–395	–	–	12
	⑤	9403.2	9403.2	9400.0	3R 268–385	–	–	7
	⑥	9330.1	9330.1	9330.0	4R 299–385	–	–	7
PiD	①	12,097.0	12,095.8	12,089.5	3R 243–387	○	–	12
	②	11,894.7	11,893.8	11,890.4	3R 243–385	○	–	12
CBD	①	13,757.9	13,756.6	13,758.3	4R 268–395	–	–	15
	②	12,647.3	12,645.3	12,644.8	4R 268–387	–	–	13
	③	10,750.1	10,751.0	10,749.7	4R 268–369	△	–	10
PSP	①	14,599.7	ND	14,600.8	4R 260–395	○	–	16
	②	13,760.4	ND	13,758.3	4R 268–395	–	–	15
	③	13,569.1	ND	13,567.3	4R 243–369	–	–	15
CBD + PSP	①	14,605.3	ND	14,600.8	4R 260–395	–	△	16
	②	13,760.7	ND	13,758.3	4R 268–395	–	△	15
	③	12,647.5	ND	12,644.8	4R 268–387	–	–	13
	④	10,751.1	ND	10,749.7	4R 268–369	△	–	10
*MAPT*	①	15,685.11 + 80x4	15,684.7	15,683.3	4R 260–406	○	○	17
	②	14,603.9	14,603.8	14,600.8	4R 260–395	○	–	16
	③	13,761.4	13,760.3	13,758.3	4R 268–395	–	–	15
	④	12,647.7	12,647.3	12,644.8	4R 268–387	–	–	13
	⑤	11,594.0	11,596.2	11,592.2	4R 260–369	○	–	12
	⑥	10,751.5	10,751.2	10,749.7	4R 268–369	–	–	10

### AD tau

MALDI-TOF MS analysis of trypsin-resistant tau from AD brains without alkaline phosphatase treatment gave signals corresponding to molecular masses of 10,517.1 (③) and 9403.2 (⑤), which match the calculated values of residues 268–395 and 268–385 of human 3R tau isoforms, and molecular masses of 10,447.2 (④) and 9330.1 (⑥) which match the calculated values of residues 299–395 and 299–385 of human 4R tau isoforms (Suppl Fig. 3a). Mass analysis of the trypsin-resistant tau after dephosphorylation gave signals corresponding to molecular masses of 11,597.7 (①), 10,517.3 (③) and 9403.2 (⑤), which match the calculated values of residues 268–406, 268–395 and 268–385 of human 3R tau isoforms, and molecular masses of 11,526.1 (②), 10,448.3 (④), and 9330.1 (⑥), which match the calculated values of residues 299–406, 299–395 and 299–385 of human 4R tau isoforms, respectively (Suppl Fig. 3a). Signals ① and ② were detected only when the fragments were dephosphorylated by alkaline phosphatase, suggesting that these fragments were phosphorylated. Protein sequence analysis of the three major bands at 7, 12 and 15 kDa from AD brains revealed that all these bands contained two fragments of both 3R and 4R tau isoforms with the N-terminal sequences, HQPGGGKVQIVY (268–279 of 3R tau) and HVPGGGSVQIVY (299–310 of 4R tau) (Suppl Fig. 3b). Thus, these 7, 12 and 15 kDa trypsin-resistant fragments consist of polypeptides with the same N-terminus, but different C-termini. These results are consistent with previous findings by Jakes et al. [20] and in our laboratory [18]. To further confirm the peptide identification, we performed LC/MS/MS analysis of V8 digests of these fragments. Mass analysis showed signals corresponding to tau peptides 392–406 (IVYKSPVVSGDTSPR), 388–395 (HGAEIVYK) and 375–385 (THKLTFRENAK) (Suppl Fig. 3b), strongly suggesting that the 7, 12 and 15 kDa AD tau bands represent tau fragments of 268–385, 268–395 and 268–406 derived from 3R tau isoforms, and 299–395, 299–385, and 299–406 derived from 4R tau isoforms (Fig. 4).

### PiD tau

MALDI-TOF MS analysis of the trypsin-resistant PiD tau gave signals corresponding to molecular masses of 12097.0 (①) and 11894.7 (②), which match the calculated values of residues 243–387 and residues 243–385 of human 3R tau isoforms, respectively (Table 2 and Suppl Fig. 4a). Alkaline phosphatase treatment had no significant effect. Protein sequence analysis of the major 12 kDa band revealed that the N-terminal sequence was LQTAPVPMPDLK, corresponding to residues 243–254 of 3R tau isoforms (Suppl Fig. 4b). LC/MS/MS analysis of V8 digests of the fragments gave signals corresponding to tau peptides 375–385 (THKLTFRENAK) and 375–387 (THKLTFRENAKAK) (Suppl Fig. 4b). These results strongly suggest that the 12 kDa PiD tau band represents tau fragments of 243–387 and 243–385 derived from 3R tau isoforms (Fig. 4).

### CBD tau

MALDI-TOF MS analysis of the CBD tryptic fragments gave signals corresponding to molecular masses of 13,757.9 (①), 12,647.3 (②), 10,750.1 (③), which match the calculated values of residues 268–395, 268–387 and 268–369 of human 4R tau isoforms, respectively (Table 2 and Suppl Fig. 5a). Alkaline phosphatase treatment had no significant effect (data not shown). Protein sequence analysis of 10, 13 and 15 kDa CBD tau revealed an N-terminal sequence, HQPGGGK, that corresponded to residues 268–274 of 4R tau (Suppl Fig. 5b). LC/MS/MS analysis of V8 digests gave signals corresponding to tau peptides 388–395 (HGAEIVYK), 375–387 (THKLTFRENAKAK) and 359–369 (NITHVPGGGNK) (Suppl Fig. 5b). These results strongly suggest that the 10, 13 and 15 kDa CBD bands represent tau fragments of 268–369, 268–387 and 268–395 derived from 4R tau isoforms, respectively (Fig. 4).

### PSP tau

MALDI-TOF MS analysis gave signals corresponding to molecular masses of 14,599.7 (①), 13,760.4 (②) and 13,569.1 (③), which match the calculated values of residues 260–395, 268–395 and 243–369 of human 4R tau isoforms, respectively (Table 2 and Suppl Fig. 6a). Because recovery of trypsin-resistant PSP tau was low, we could not carry out protein sequence analysis. However, LC/MS/MS analysis of V8 digests showed signals corresponding to tau peptides 388–395 (HGAEIVYK) and 359–369 (NITHVPGGGNK) (Suppl Fig. 6b). These results suggest that the trypsin-resistant PSP tau fragments may represent 260–395, 268–395 and 243–369 of 4R tau isoforms. We also analyzed trypsin-resistant tau from one case with a complication of CBD and PSP. The mass analyses detected a mixture of the tau fragments detected in CBD and in PSP (Suppl Fig. 7a, b).

### Tau in *MAPT*

**Fig. 5:**
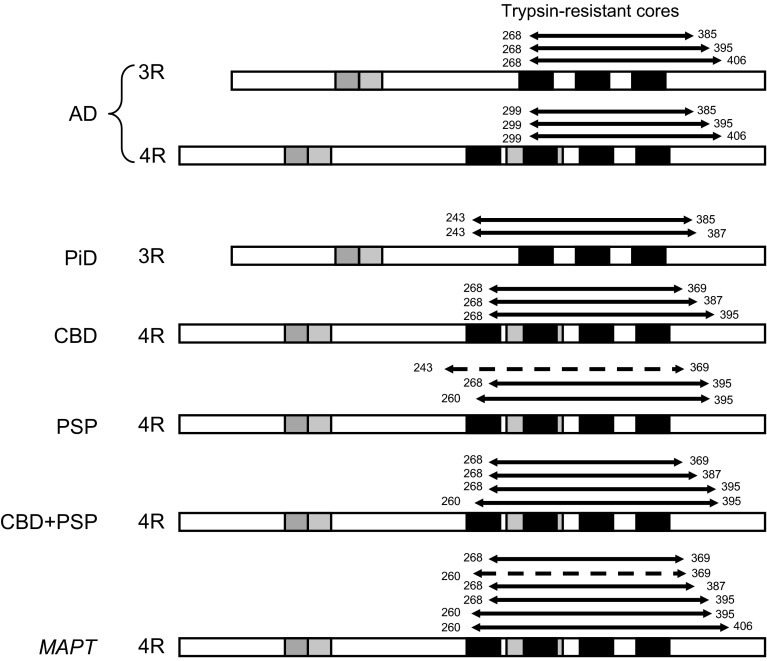
Summary of trypsin-resistant regions of pathological tau from tauopathy brains. The major and minor tau fragments identified as trypsin-resistant tau in these tauopathies are indicated by *solid* and *broken lines*, respectively. Trypsin-resistant cores of AD-tau are distinct from the others in terms of both the isoforms and regions. AD tau is composed of equimolar amounts of both 3R tau and 4R tau isoforms and the cores are localized to start from the middle of the 1st repeat of 3R tau and the 2nd repeat of 4R tau, which are different from those of the other tauopathies composed of only 3R or only 4R tau isoforms (20–30 amino acid residues slide toward the C-terminus)

MALDI-TOF mass analysis of *MAPT* tau with or without alkaline phosphatase treatment gave 6 signals corresponding to molecular masses of 15,684.7 (①), 14,603.8 (②), 13,760.3 (③), 12,647.3 (④), 11,596.2 (⑤) and 10,751.2 (⑥), which match the calculated values of residues 268–369, 260–369, 268–387, 268–395, 260–395 and 260–406 of human 4R tau isoforms, respectively (Table 2 and Suppl Fig. 8a, b). The mass signal of 15,684.7 (①) was decreased after dephosphorylation suggesting that the mass signal of 16,005.1 (①’) may represent the mass 15,684.7(①) signal plus 4 phosphoric acid moieties (80 × 4 = 320); this matches the calculated values of residues 260–406 of human 4R tau. As in the case of PSP tau, we could not detect the N-terminal sequence of trypsin-resistant tau. Therefore, we tried to assign the fragments based on the results of mass analyses and immunoblotting. This 17 kDa band was immunopositive for PHF1, which is consistent with phosphorylation of S396/404. LC/MS/MS analysis of V8 digests showed signals corresponding to tau peptides 392–406 (IVYKSPVVSGDTSPR), 388–395 (HGAEIVYK), 375–387 (THKLTFRENAKAK) and 359–369 (NITHVPGGGNK) (Suppl Fig. 8b), strongly suggesting that residues 260–406, 260–395, 268–395, 268–387, 260–369 and 268–369 of 4R tau comprise the 17, 16, 15, 13, 12 and 10 kDa trypsin-resistant fragments of *MAPT*, respectively. The trypsin-resistant core regions of pathological tau identified in this study are illustrated schematically in Fig. 5.

### Electron microscopy of sarkosyl-insoluble tau fibrils in tauopathies

**Fig. 6:**
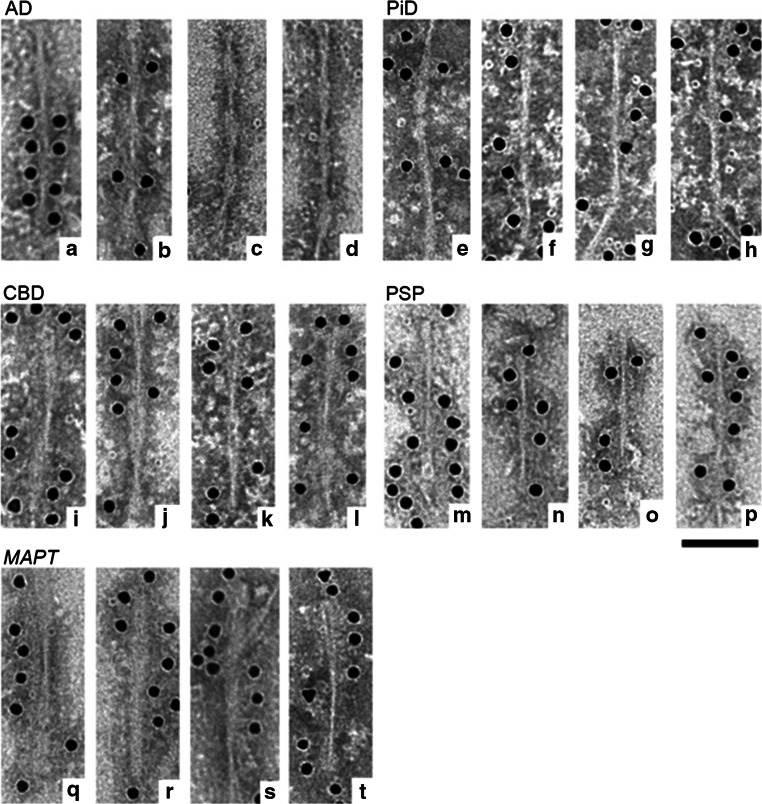
Electron microscopy of sarkosyl-insoluble tau from tauopathy brains. Electron micrographs of PHFs from AD brains, which were positive for T46 (**a**, **b**) which were labeled by secondary antibody conjugated to 15-nm gold particles. Similar PHFs were observed after trypsin digestion (**c**, **d**). T46-positive SFs were observed in PiD (**e**–**h**). Twisted filaments in CBD were positive for T46 (**i**–**l**). In PSP thin twisted filaments were labeled with T46 (**m**–**p**). In *MAPT*, ribbon-like filaments similar to those in CBD were observed (**q**–**t**)

Immunoelectron microscopy of the sarkosyl-insoluble fractions from these tauopathy brains was performed to investigate the relationship between the trypsin-resistant cores and the morphology of the tau fibrils. Representative pictures of the tau fibrils are shown in Fig. 6. In the tau fractions from AD brains, many fibrils with PHF morphology (10–20 nm in diameter with 80 nm periodicity) were observed. Some were immunopositive for T46 antibody (Fig. 6a, b), but unlabeled PHFs were also observed. Similar structures were also observed after the trypsin digestion (Fig. 6c, d). In PiD, straight filaments (13–16 nm in diameter) labeled with T46 were observed (Fig. 6e–h). Twisted filaments (10–25 nm in diameter with 140–180 periodicity) (Fig. 6i–l) in CBD and similar twisted ribbon-like filaments (10–25 nm diameter with broader periodicity than those of CBD) were seen in *MAPT* intronic mutations (Fig. 6q–t). On the other hand, thinner twisted filaments (7–15 nm in diameter with 80–150 nm periodicity) were observed in PSP (Fig. 6m–p). Similar filamentous structures were observed after these trypsin digestion, although the numbers were significantly reduced (data not shown).

## Discussion

Tauopathies are neurodegenerative disorders characterized by accumulation of abnormal tau proteins in neuronal and/or glial cells within the brain. In this study, we examined sarkosyl-insoluble and trypsin-resistant fragments of abnormal tau from brains of patients with AD, PiD, CBD, PSP, and intronic *MAPT* mutations, and found that the core units of the tau aggregates were composed of different tau repeat regions located between residues 243–406, indicating that the conformations of the aggregates are disease-specific. These patterns of trypsin-resistant tau are reminiscent of the proteinase-K-resistant bands in human prion disorders, and suggest that tauopathies may be caused by accumulation of toxic tau ‘prions’ in the brain. A wide range of phenotypic variation has been found in prion diseases, and has been attributed to the existence of distinct “strains” of the agent or prion, and different mutations and polymorphisms of prion protein in the host [12, 30]. Furthermore, the differences in the abnormal prion proteins in brains of affected patients, which are distinguished by distinct proteinase-K-resistant banding patterns, correlate with the disease phenotypes [39].

In this study, we show that the banding patterns of C-terminal fragments of tau are different among different tauopathies, and the trypsin-resistant band patterns are also distinct among the diseases. Our previous study demonstrated that PSP and CBD differ in the pattern of tau fragments on immunoblots of sarkosyl-insoluble brain extracts [1]. The present study confirmed and extended these findings indicating that the differences may be related to the protease resistance of these pathological tau proteins. Employing analysis of sarkosyl-insoluble tau banding patterns using antibodies against tau C-terminus, tauopathies can be biochemically classifiable into at least four types, PiD, PSP, CBD and AD. In addition, we have shown that pathological 4R tau in *MAPT* intron mutation cases can be distinguishable from that in CBD and PSP cases by the analysis of the C-terminal fragments and the trypsin-resistant tau. These biochemical characterizations of pathological tau provide evidence that these diseases with distinct histopathologies are different disease entities with distinct tau species. It is possible that the detection of protease-resistant tau bands may also be useful for biochemical classification of other tauopathies with distinct histopathologies, such as AGD, globular glial tauopathy and tangle only dementia, warranting further investigation.

Recent studies using cellular and animal models have demonstrated that intracellular pathological proteins, including tau protein, propagate through neuronal networks via prion-like mechanisms [8, 24–26]. Injection of brain homogenates from patients with AD, PiD, PSP, CBD and AGD into Tg-mice overexpressing human 4R tau induced tau pathologies that were similar to those of the tauopathies [7]. These findings strongly suggest that a structurally distinct pathological tau protein is involved in each disease, and has the ability to convert normal tau protein into its own abnormal form (seed/template-dependent prion-like conversion). It is likely that the protease-resistant bands represent the core regions of tau fibrils, and thus, it is plausible that the differences in these bands in different diseases reflect different conformations of the tau assemblies. Indeed, tau accumulated in AD brains has a unique abnormal structure, i.e., paired helical filaments (PHF), and the microtubule-binding regions of both 3R and 4R tau form the core structure of PHF [19]. The sequences of trypsin-resistant tau identified in this study in various tauopathies are also located in microtubule-binding regions and the adjacent C-terminal region, though the N- and C-terminal sequences and the sequence lengths differ slightly among the diseases. These findings suggest that the fibril cores are somewhat different among the diseases and the different structures of the assembled tau proteins may determine the characteristic tau pathologies. The differences in sensitivity to conventional staining with dyes or immunostaining may be due to these distinct structures [36].

As shown in Table 2, little or no differences were detected in the post-translational modifications of the trypsin-resistant tau fragments between the diseases. This is also similar to that seen in prion diseases, where no significant difference of modification was identified between normal prion protein and the abnormal form, whereas the conformations between the two are different. In tauopathies, the carboxy-third of tau is shown to comprise the protease-resistant building block of paired helical filaments in AD. Similar, but distinctly characteristic, C-terminal tau fragments were detected as the trypsin-resistant bands in this study. Tau mutations causing tauopathies are mostly concentrated in the microtubule-binding and C-terminal regions. In contrast, most post-translational modifications, such as phosphorylation, are identified surrounding the microtubule-binding repeat regions, namely in the trypsin-resistant core regions of sarkosyl-insoluble tau, suggesting that these modifications, especially the phosphorylation, may occur following tau assembly and conformational changes.

It has been reported that Ser 262 is less phosphorylated in PiD than in other tauopathies [3, 31]. This may be because the epitope is buried in the cores of the tau fibrils in PiD (243–385, 243–387), and therefore may be hardly accessible for phosphorylation by kinases after assembly, whereas the epitope is largely located outside the fibril cores in other tauopathies (ex. 268–406 in AD, 267–395 in PSP, CBD). Electron-microscopic observations revealed that tau is deposited as filamentous or fibrous forms with distinct morphologies among the diseases: PHF in AD, SF in PiD, twisted fibrils in CBD, and twisted ribbons in *MAPT* [4, 14]. Indeed, PHFs with 10–20 nm diameters in AD, SF with 13–16 nm in PiD, twisted filaments with 10–25 nm in CBD, thin twisted filaments with 7–15 nm in diameter in PSP and twisted ribbon-like filaments with 10–25 nm diameter in *MAPT* were observed in this study (Fig. 6). Thus, it seems reasonable to speculate that the C-terminal fragments and trypsin-resistant cores of these tau may be related to the fibril structures, especially the diameter and periodicity of the fibrils. We found 7, 10 and 15 kDa trypsin-resistant fragments in AD, where tau is assembled into highly ordered PHF 10–20 nm in diameter, while a ~12 kDa fragment was detected in PiD, which exhibits SF 13–16 nm in diameter. Several 10–25 kDa trypsin-resistant fragments were detected in CBD and *MAPT*, in which unique ribbon-like filamentous structures with 10–25 nm diameter were observed, whereas thinner twisted filamentous structures were observed in PSP which shows the unique ~33 kD CTFs and 10–16 kD trypsin-resistant bands. Although it remains to be seen how the tau molecules are integrated into the fibrils, the above results suggest that various homodimers of tau microtubule-binding regions of 3R or 4R tau isoforms and heterodimers of 3R/4R tau isoforms in the case of AD may be formed and assembled with a constant periodicity. Predicted models of tau fibril cores in the various tauopathies based on an antiparallel association are illustrated in Suppl Fig. 9.

Another important issue is the minimal unit of abnormal tau protein that serve as a template for prion-like propagation. Although this remains to be clarified in the case of prion protein, it is strongly suggested that the dimer may be the minimal unit in tauopathies, because an equimolar ratio of 3R and 4R tau isoforms is integrated into the unique filamentous structure (PHF) in AD, whereas fibrils composed of only 3R tau or only 4R tau are deposited in other tauopathies. Our results suggest that it may be reasonable to classify the neuropathological subtypes of tauopathies biochemically in terms of deposition of 3R and/or 4R tau isoforms, the banding patterns of C-terminal fragments, and the protease-resistant domains. The results obtained in this study may also contribute to the development of PET probes specific for the characteristic structure of abnormal tau in each of the tauopathies.

## Electronic supplementary material

Supplementary material 2 (PPT 2455 kb)
