# Supplementary material for: Biochemical classification of tauopathies by immunoblot, protein sequence and mass spectrometric analyses of sarkosyl-insoluble and trypsin-resistant tau
Source: Acta Neuropathol. 2015 Nov 4;131:267–80. doi: 10.1007/s00401-015-1503-3 (PMC4713716; doi:10.1007/s00401-015-1503-3)
Supplement: Supplementary file 1 — Supplementary material 2 (PPT 2455 kb) [file 401_2015_1503_MOESM1_ESM.ppt]

## Slide 1
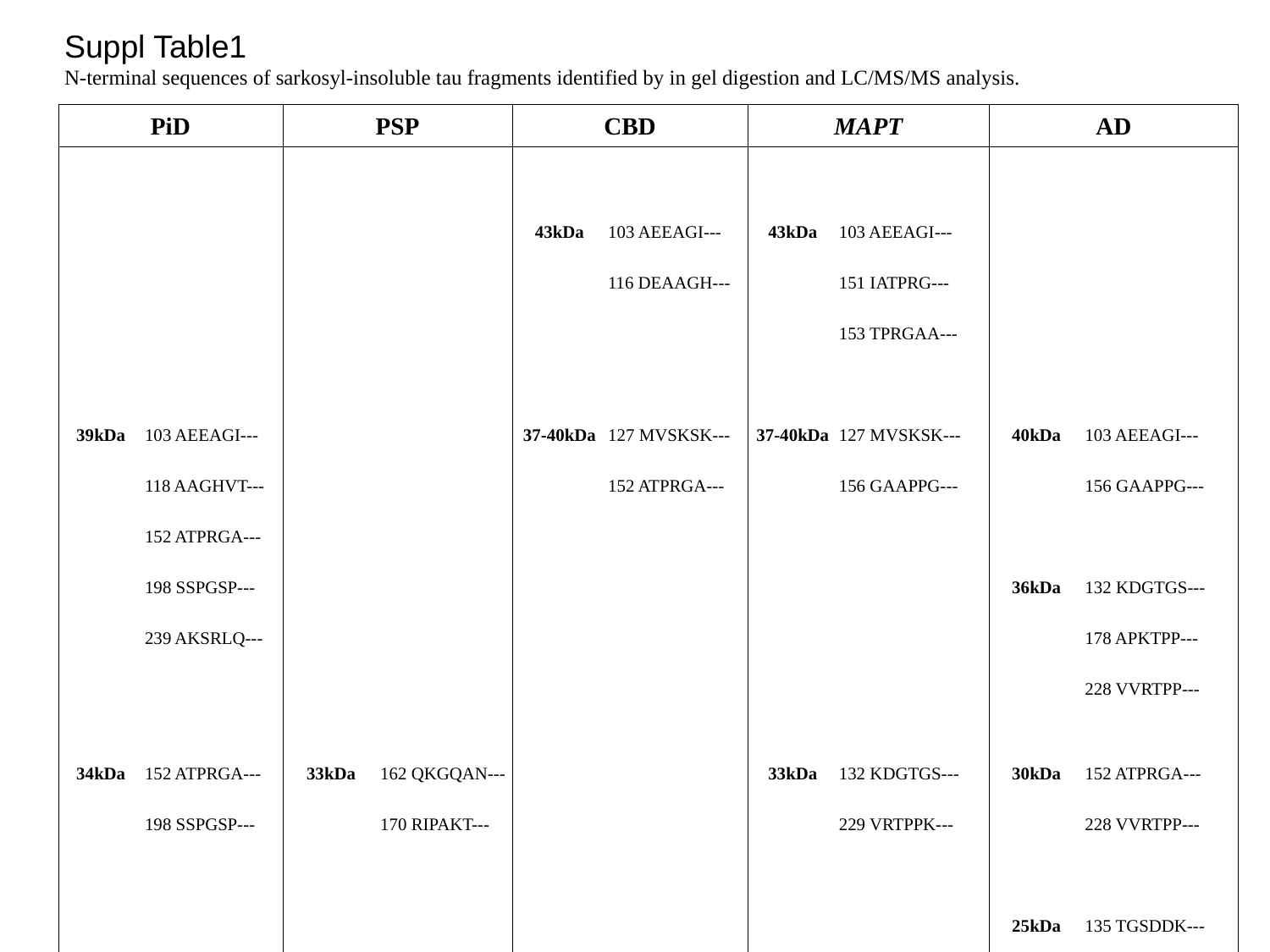

Suppl Table1
N-terminal sequences of sarkosyl-insoluble tau fragments identified by in gel digestion and LC/MS/MS analysis.
| PiD | | PSP | | CBD | | MAPT | | AD | |
| --- | --- | --- | --- | --- | --- | --- | --- | --- | --- |
| | | | | | | | | | |
| | | | | 43kDa | 103 AEEAGI--- | 43kDa | 103 AEEAGI--- | | |
| | | | | | 116 DEAAGH--- | | 151 IATPRG--- | | |
| | | | | | | | 153 TPRGAA--- | | |
| | | | | | | | | | |
| 39kDa | 103 AEEAGI--- | | | 37-40kDa | 127 MVSKSK--- | 37-40kDa | 127 MVSKSK--- | 40kDa | 103 AEEAGI--- |
| | 118 AAGHVT--- | | | | 152 ATPRGA--- | | 156 GAAPPG--- | | 156 GAAPPG--- |
| | 152 ATPRGA--- | | | | | | | | |
| | 198 SSPGSP--- | | | | | | | 36kDa | 132 KDGTGS--- |
| | 239 AKSRLQ--- | | | | | | | | 178 APKTPP--- |
| | | | | | | | | | 228 VVRTPP--- |
| | | | | | | | | | |
| 34kDa | 152 ATPRGA--- | 33kDa | 162 QKGQAN--- | | | 33kDa | 132 KDGTGS--- | 30kDa | 152 ATPRGA--- |
| | 198 SSPGSP--- | | 170 RIPAKT--- | | | | 229 VRTPPK--- | | 228 VVRTPP--- |
| | | | | | | | | | |
| | | | | | | | | 25kDa | 135 TGSDDK--- |
| | | | | | | | | | 226 VAVVRT--- |
| | | | | | | | | | 228 VVRTPP--- |
| | | | | | | | | | |
| 21kDa | 241 SRLQTA--- | 22kDa | 199 SPGSPG--- | 22kDa | 187 EPPKSG--- | 22kDa | 194 RSGYSS--- | 22kDa | 226 VAVVRT--- |
| | 245 TAPVPM--- | | | | 239 AKSRLQ--- | | 232 PPKSPS--- | | 228 VVRTPP--- |
| | | | | | 245 TAPVPM--- | | 245 TAPVPM--- | | 229 VRTPPK--- |
| | | | | | | | | | 245 TAPVPM--- |
| | | | | | | | | | 247 PVPMPD--- |
| | | | | | | | | | 297 IKHVPG--- |
| | | | | | | | | | |
| | | | | | | | | 19kDa | 198 SSPGSP--- |
| | | | | | | | | | 239 AKSRLQ--- |
| | | | | | | | | | 245 TAPVPM--- |
| | | | | | | | | | 304 GSVQIV--- |
| | | | | | | | | | 305 SVQIVY--- |
| | | | | | | | | | 306 VQIVYK--- |
| | | | | | | | | | |

## Slide 2
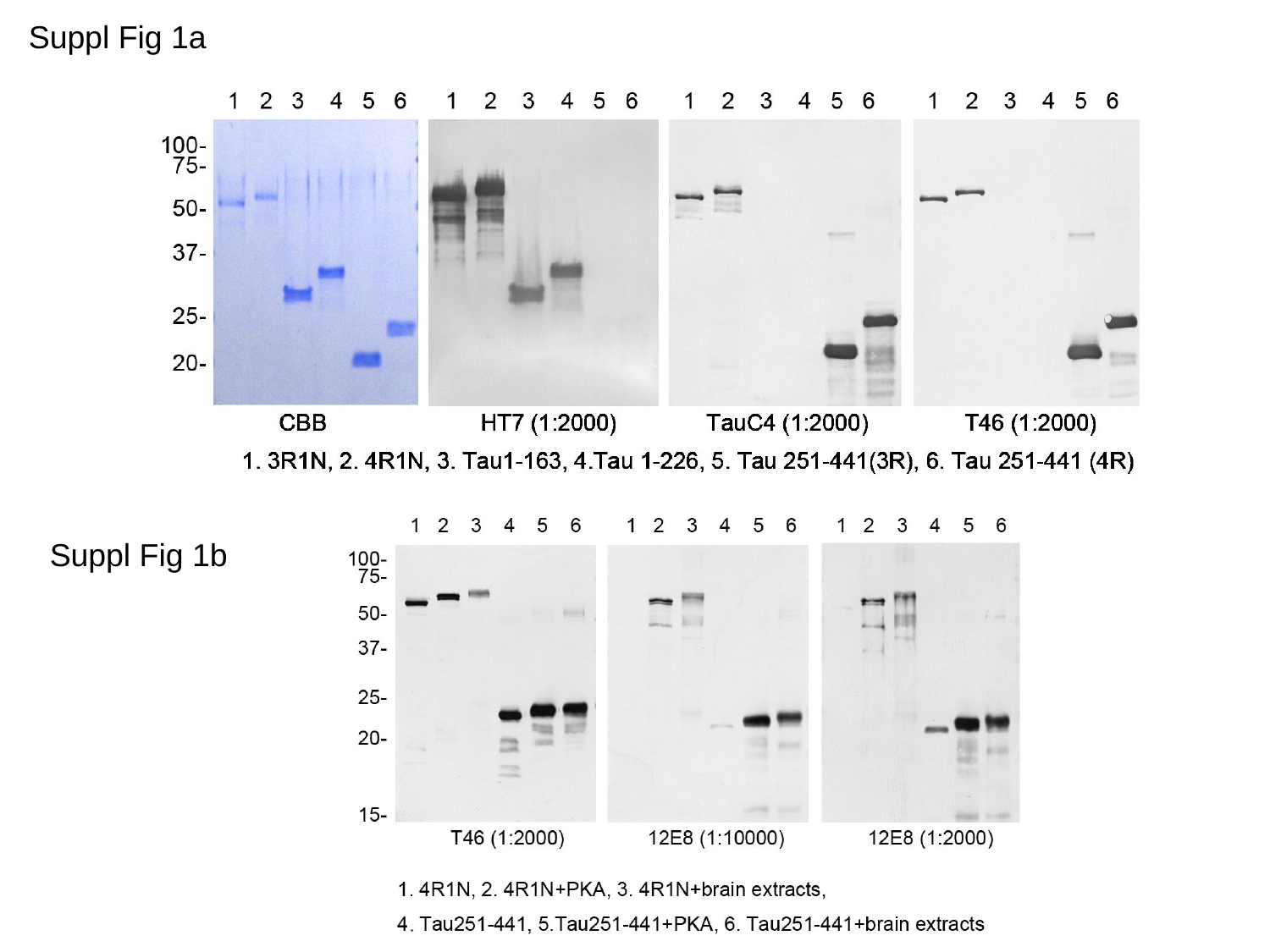

Suppl Fig 1a
Suppl Fig 1b

## Slide 3
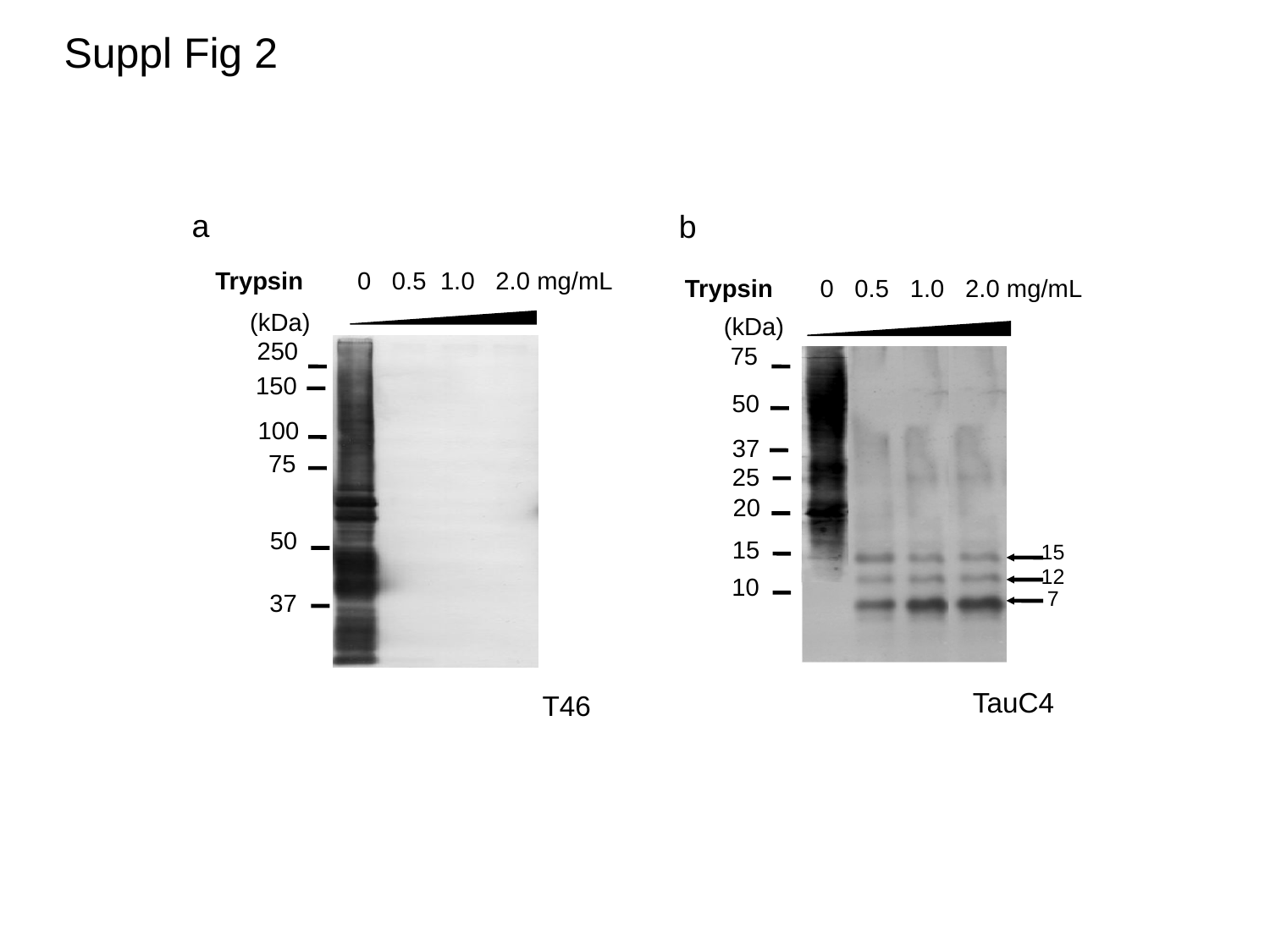

Suppl Fig 2
a
b
Trypsin 0 0.5 1.0 2.0 mg/mL
Trypsin 0 0.5 1.0 2.0 mg/mL
(kDa)
 250
(kDa)
 75
150
50
100
37
75
25
20
50
15
15
12
10
7
37
TauC4
T46

## Slide 4
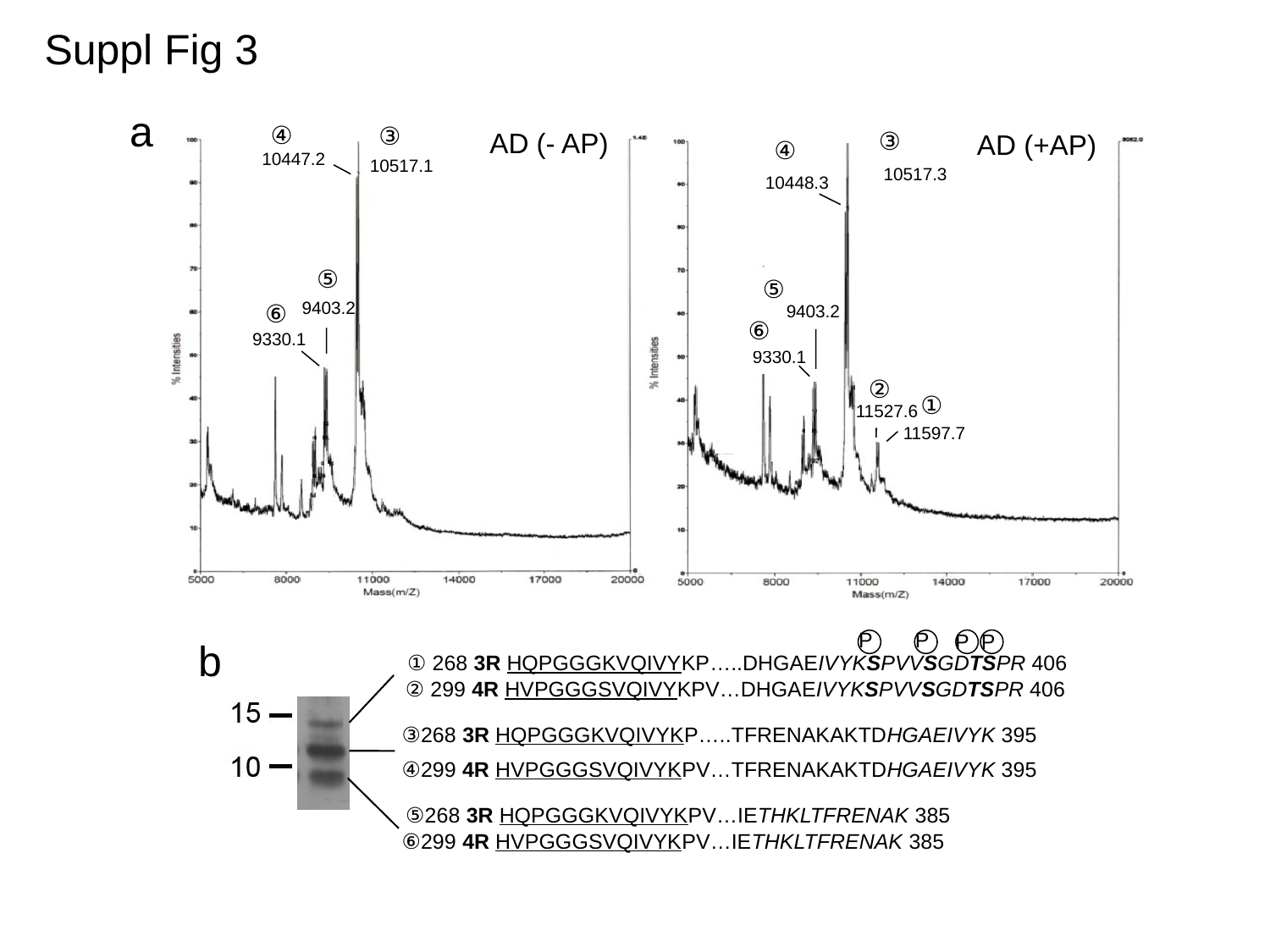

Suppl Fig 3
a
 ④
 ③
AD (- AP)
AD (+AP)
 ③
 ④
10447.2
10517.1
10517.3
10448.3
 ⑤
 ⑤
9403.2
 ⑥
9403.2
 ⑥
9330.1
9330.1
 ②
 ①
11527.6
11597.7
P
P
P
P
b
① 268 3R HQPGGGKVQIVYKP…..DHGAEIVYKSPVVSGDTSPR 406
② 299 4R HVPGGGSVQIVYKPV…DHGAEIVYKSPVVSGDTSPR 406
③268 3R HQPGGGKVQIVYKP…..TFRENAKAKTDHGAEIVYK 395
④299 4R HVPGGGSVQIVYKPV…TFRENAKAKTDHGAEIVYK 395
⑤268 3R HQPGGGKVQIVYKPV…IETHKLTFRENAK 385
⑥299 4R HVPGGGSVQIVYKPV…IETHKLTFRENAK 385

## Slide 5
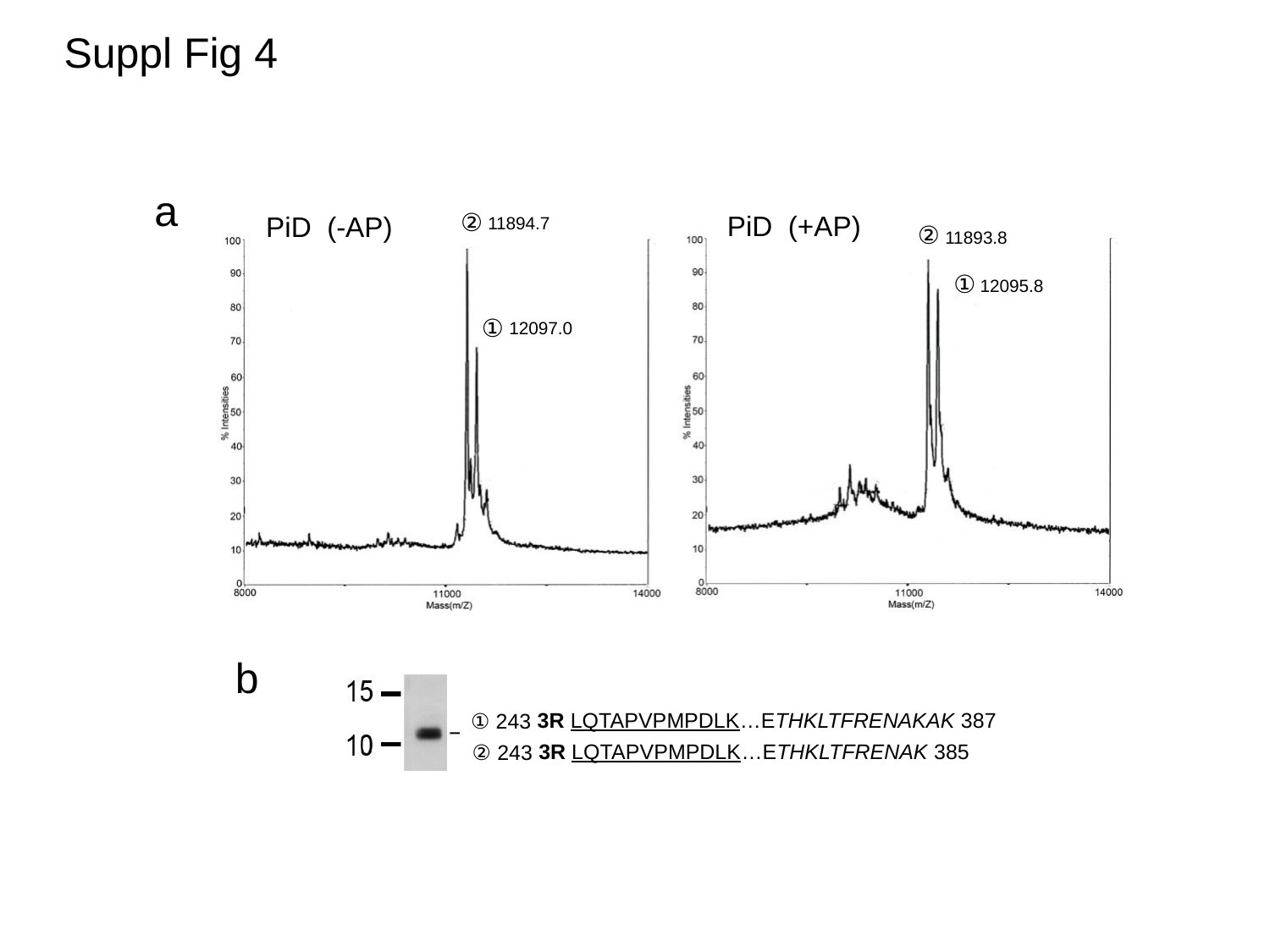

Suppl Fig 4
a
 ②
11894.7
 ②
11893.8
 ①
12095.8
 ①
12097.0
PiD (+AP)
PiD (-AP)
b
① 243 3R LQTAPVPMPDLK…ETHKLTFRENAKAK 387
② 243 3R LQTAPVPMPDLK…ETHKLTFRENAK 385

## Slide 6
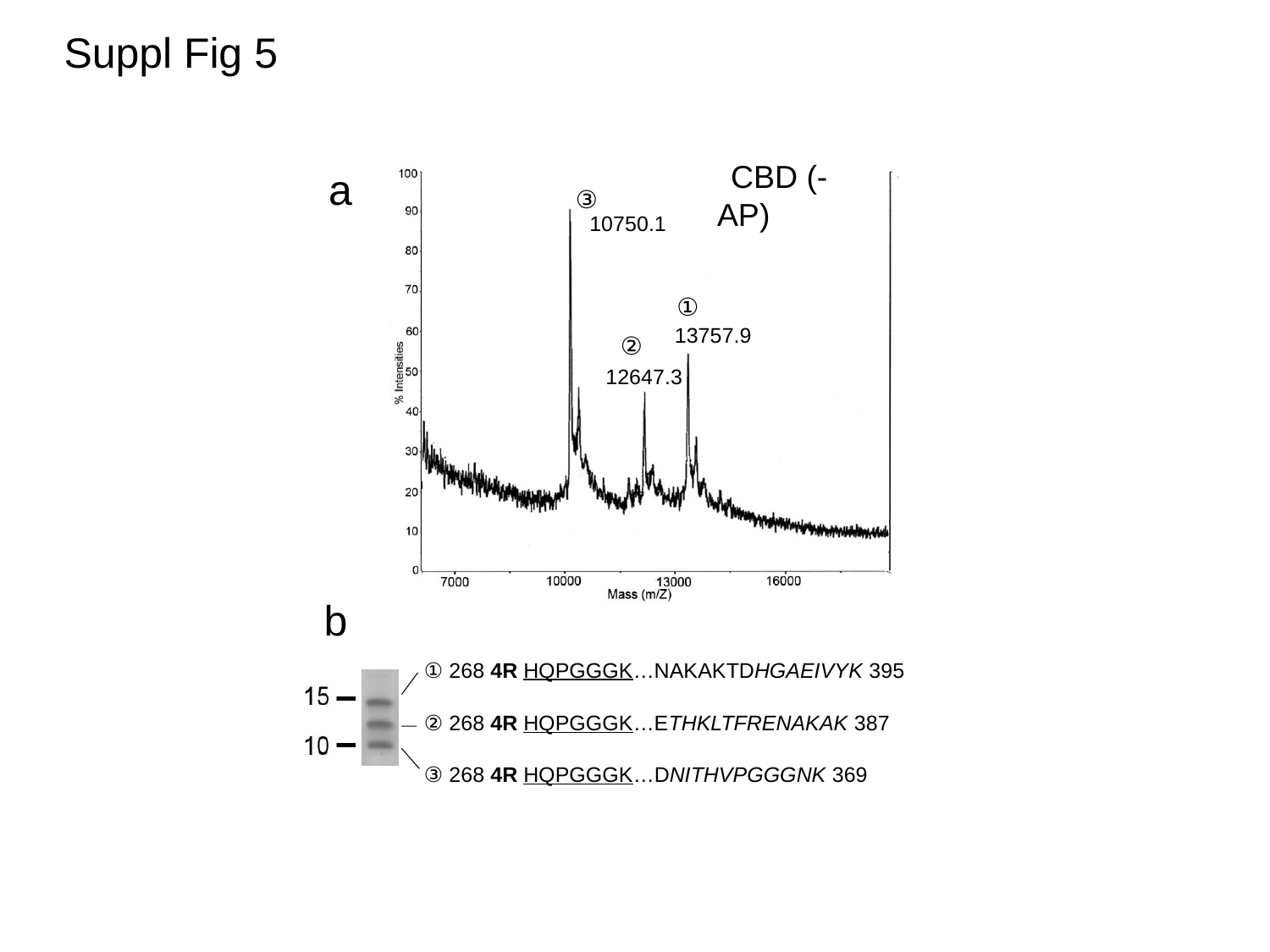

Suppl Fig 5
CBD (- AP)
 ③
10750.1
 ①
13757.9
 ②
12647.3
a
b
① 268 4R HQPGGGK…NAKAKTDHGAEIVYK 395
② 268 4R HQPGGGK…ETHKLTFRENAKAK 387
③ 268 4R HQPGGGK…DNITHVPGGGNK 369

## Slide 7
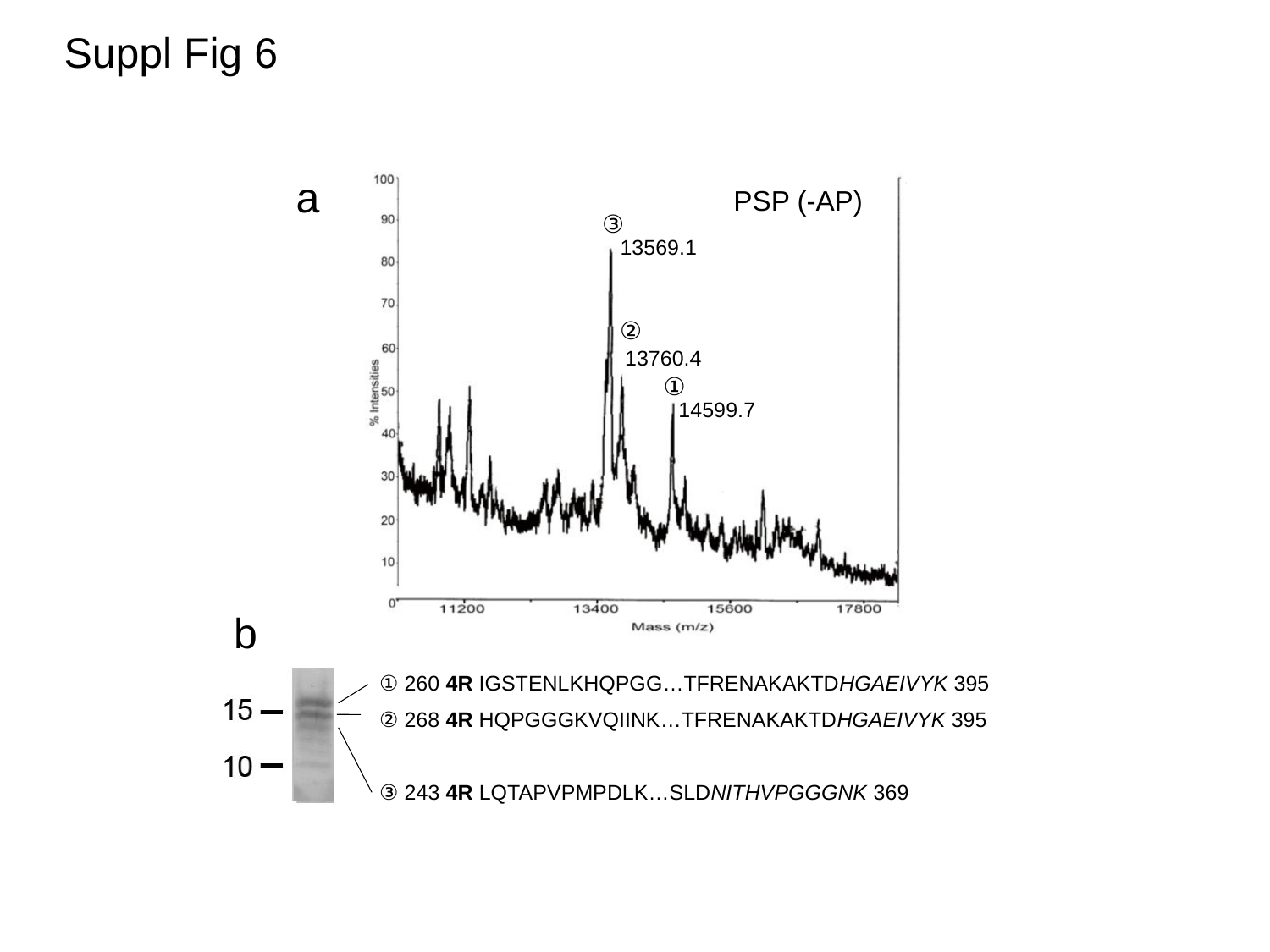

Suppl Fig 6
a
PSP (-AP)
 ③
13569.1
 ②
13760.4
 ①
14599.7
b
① 260 4R IGSTENLKHQPGG…TFRENAKAKTDHGAEIVYK 395
② 268 4R HQPGGGKVQIINK…TFRENAKAKTDHGAEIVYK 395
③ 243 4R LQTAPVPMPDLK…SLDNITHVPGGGNK 369

## Slide 8
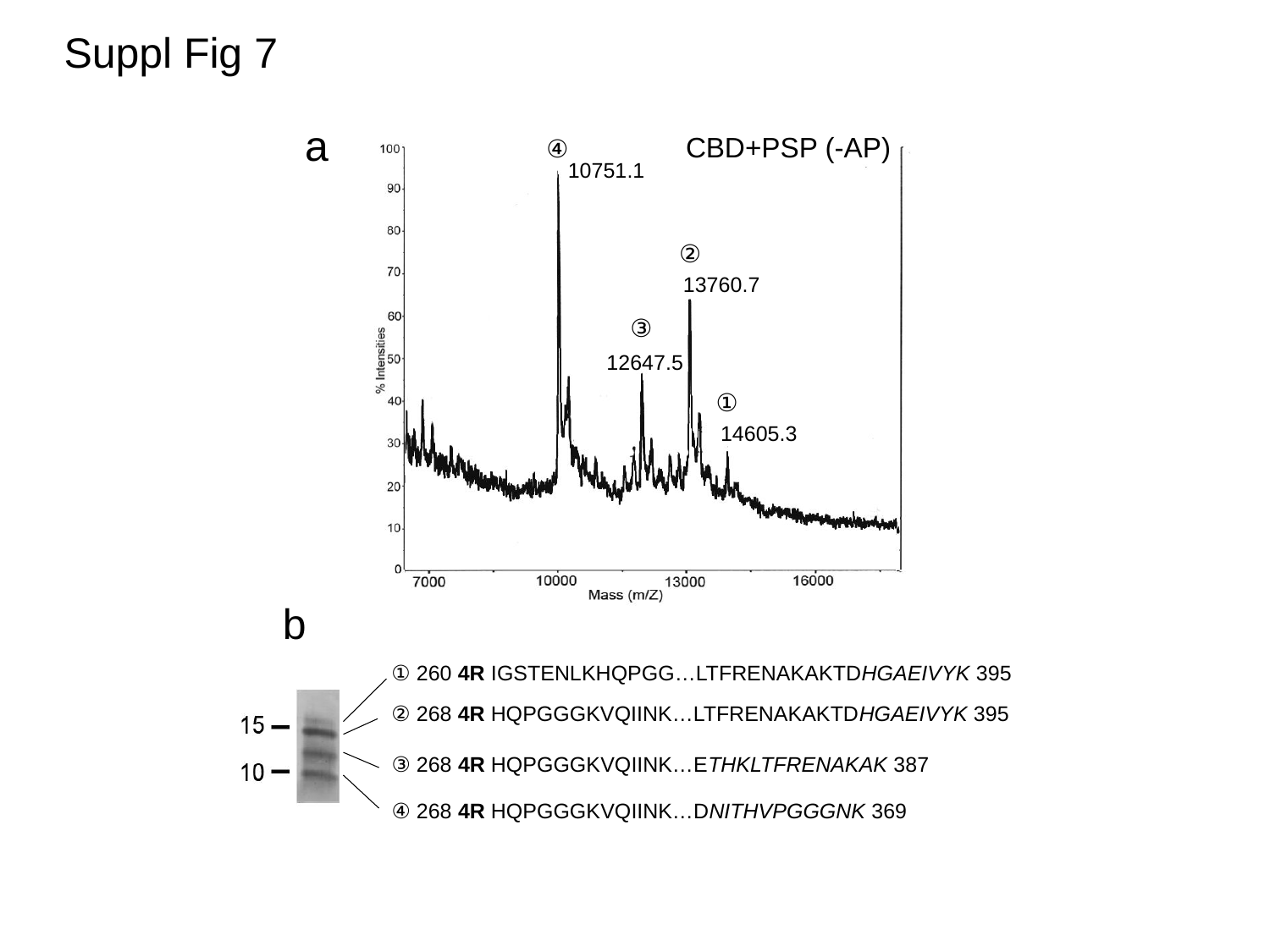

Suppl Fig 7
a
CBD+PSP (-AP)
 ④
10751.1
 ②
13760.7
 ③
12647.5
 ①
14605.3
b
① 260 4R IGSTENLKHQPGG…LTFRENAKAKTDHGAEIVYK 395
② 268 4R HQPGGGKVQIINK…LTFRENAKAKTDHGAEIVYK 395
③ 268 4R HQPGGGKVQIINK…ETHKLTFRENAKAK 387
④ 268 4R HQPGGGKVQIINK…DNITHVPGGGNK 369

## Slide 9
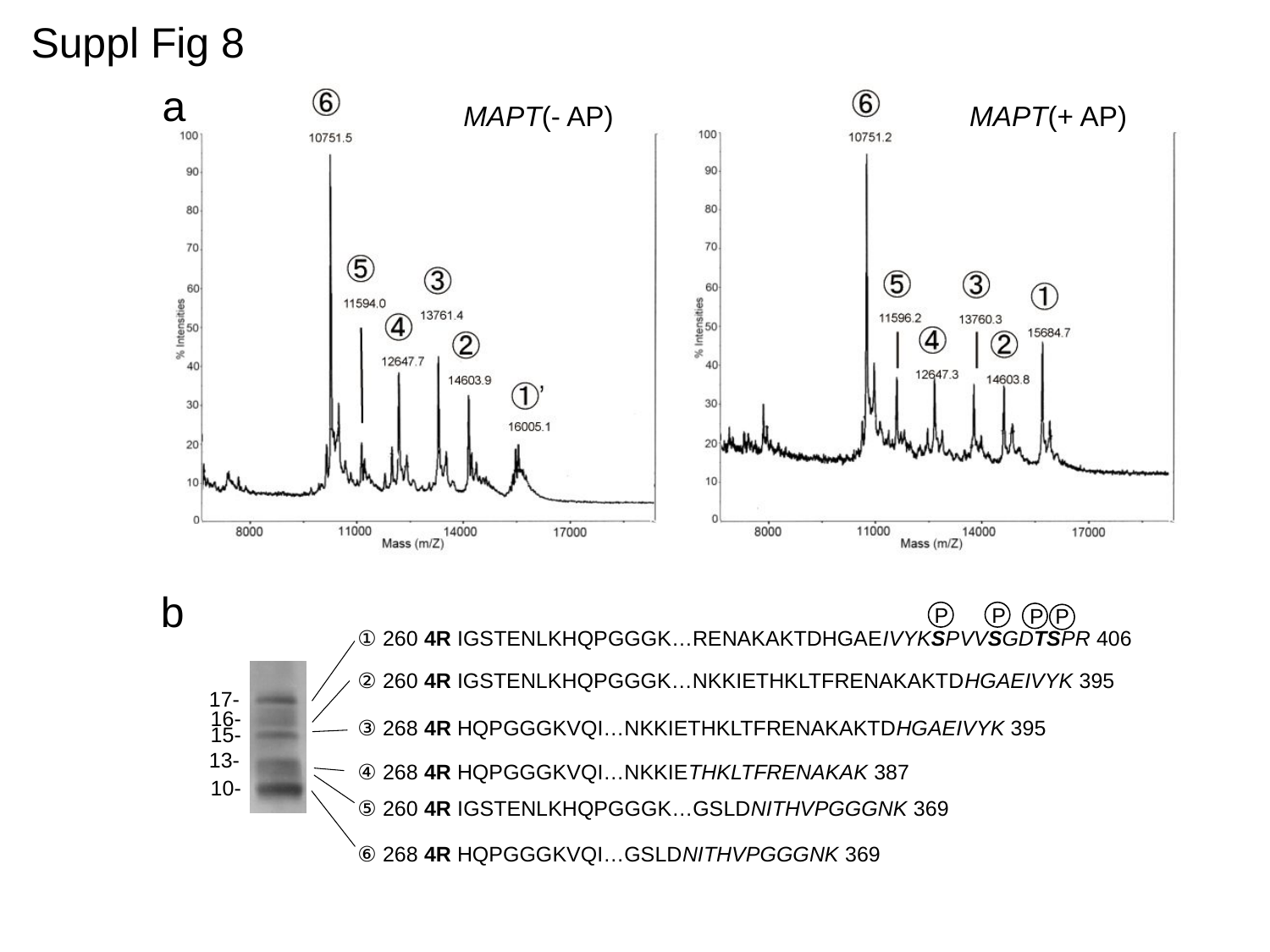

Suppl Fig 8
a
MAPT(- AP)
MAPT(+ AP)
b
P
P
P
P
① 260 4R IGSTENLKHQPGGGK…RENAKAKTDHGAEIVYKSPVVSGDTSPR 406
② 260 4R IGSTENLKHQPGGGK…NKKIETHKLTFRENAKAKTDHGAEIVYK 395
17-
16-
15-
13-
10-
③ 268 4R HQPGGGKVQI…NKKIETHKLTFRENAKAKTDHGAEIVYK 395
④ 268 4R HQPGGGKVQI…NKKIETHKLTFRENAKAK 387
⑤ 260 4R IGSTENLKHQPGGGK…GSLDNITHVPGGGNK 369
⑥ 268 4R HQPGGGKVQI…GSLDNITHVPGGGNK 369

## Slide 10
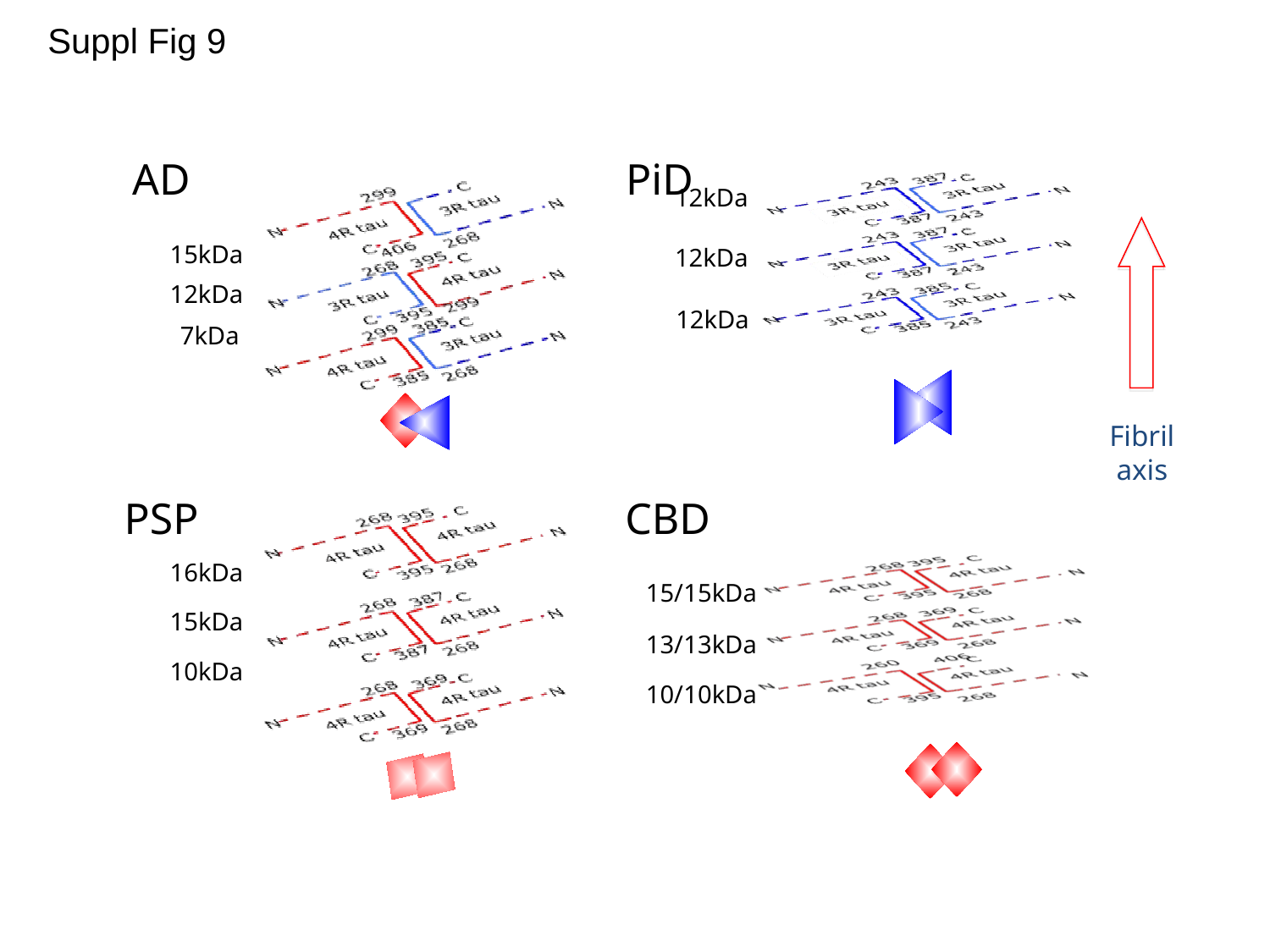

Suppl Fig 9
AD
 PiD
12kDa
12kDa
12kDa
15kDa
12kDa
7kDa
Fibril
axis
PSP
CBD
16kDa
15/15kDa
15kDa
13/13kDa
10kDa
10/10kDa
